# Depression in Intimate Partner Violence Victims in Slovenia: A Crippling Pattern of Factors Identified in Family Practice Attendees

**DOI:** 10.3390/ijerph15020210

**Published:** 2018-01-26

**Authors:** Nena Kopčavar Guček, Polona Selič

**Affiliations:** Department of Family Medicine, Faculty of Medicine, University of Ljubljana, Poljanski nasip 58, 1000 Ljubljana, Slovenia; nenagucek@gmail.com

**Keywords:** intimate partner violence, self-rated depression, mental health, family practice, sick leave, incapacity to work

## Abstract

This multi-centre cross-sectional study explored associations between prevalence of depression and exposure to intimate partner violence (IPV) at any time in patients’ adult life in 471 participants of a previous IPV study. In 2016, 174 interviews were performed, using the Short Form Domestic Violence Exposure Questionnaire, the Zung Scale and questions about behavioural patterns of exposure to IPV. Family doctors reviewed patients’ medical charts for period from 2012 to 2016, using the Domestic Violence Exposure Medical Chart Check List, for conditions which persisted for at least three years. Depression was found to be associated with any exposure to IPV in adult life and was more likely to affect women. In multivariable logistic regression modelling, factors associated with self-rated depression were identified (*p* < 0.05). Exposure to emotional and physical violence was identified as a risk factor in the first model, explaining 23% of the variance. The second model explained 66% of the variance; past divorce, dysfunctional family relationships and a history of incapacity to work increased the likelihood of depression in patients. Family doctors should consider IPV exposure when detecting depression, since lifetime IPV exposure was found to be 40.4% and 36.9% of depressed revealed it.

## 1. Introduction

Violence and mental health problems and their possible association and coexistence, have been extensively researched and published [1]. According to WHO, domestic violence should be prioritized as a public health problem, due to its prevalence as well as its consequences [2]. The associations between mental health problems and exposure to IPV in the victims and also in the perpetrators, are complex [1,3]. While substance abuse co-morbidity and a past history of violence are considered the strongest predictors of future violence, current evidence is not enough to suggest that severe mental illness can independently predict violent behaviour [4,5,6,7,8,9].

Exposure to psychological abuse was found to be more strongly associated with the prevalence of depression, anxiety, somatization, experiencing suicidal thoughts and post-traumatic stress disorder than with other types of IPV [3,8,9,10]. Women are more likely to become victims of IPV and twice as likely to become depressed [11,12]. The gender difference in the frequency of depression can be accounted for by women experiencing greater poverty, differing social roles and sex discrimination, more negative life events and violence and abuse [13]. Evidence indicates that partner abuse may contribute to depression [13]. Female victims who had suffered IPV in the past year were found to have their relative risk of depression increased by 3.26 compared to non-abused women [14]. A meta-analysis on the prevalence of mental health problems in women with a history of IPV in 1999 found that just under half of the abused women had clinical depression [15].

Abundant literature on the consequences of IPV on health has warned against undetected depression in IPV victims [16]; IPV-related depression was first discussed as a relevant health-related problem in family medicine several years ago [9], yet has never been studied as such in Slovenia. Given that the data for past IPV abuse had already been re-evaluated, showing a prevalence of exposure to all types of IPV of 17% in family medicine clinics attendees [17] and a prevalence of psychological IPV alone of approximately 10%, it was of the utmost importance for Slovenian family medicine to focus on the health-related outcomes of exposure to IPV. This study therefore aimed to explore the associations between the prevalence of depression in patients who were exposed to IPV at any time in their adult lives and those who were not; to identify the health consequences and other patient characteristics associated with exposure to IPV; and to examine whether there were any specifically gender-related issues in family clinic attendees. Since prior results showed that current health status was not associated with the current psychological IPV exposure [9], this study design covered a longer time frame when assessing IPV-related health conditions and the prevalence of depression, providing a list of health conditions that need in-depth exploration towards possible IPV exposure in family medicine attendees, i.e., chronic pain syndrome, incapacity to work, muscle inflammations (myalgia, muscle soreness or musculoskeletal pain), gastrointestinal disorders, irregularities in bowel functioning, reduced physical functioning, gynaecological disorders, genital tract infections, the state of depression and/or generalized anxiety disorder, eating and sleeping disorders, phobias and panic attacks, low self-esteem and psychosomatic disorders.

## 2. Materials and Methods

### 2.1. Participants and Procedure

Sixty-four family doctors (FDs), i.e., physicians who have finished four years of specialized training and who had already taken part in the 2012 IPV prevalence re-evaluation study [17] and participated in the 2013 Psychological IPV study [9], were invited to participate in this follow-up study in September 2016.

The 2013 study [9] was also multi-centre and cross-sectional, recruiting 960 family practice attendees aged 18 years and above, without dementia or even mild cognitive impairment, who were willing to participate. In 689 interviews with currently- or previously-partnered patients, the short form of A Domestic Violence Exposure Questionnaire and additional questions about behavioural patterns of exposure to psychological abuse in the past year were given. The FDs reviewed the medical charts of 470 patients who had been in an intimate relationship in the previous five-year period. Since we aimed to analyse solely psychological abuse, patients who had experienced psychological IPV during their lifetime but not in the past year, as well as victims of multiple types of abuse, were excluded and offered help and assistance. Using the Domestic Violence Exposure Medical Chart Check List, data on the patients’ lives, physical, sexual and reproductive and psychological health status, as well as sick leave, hospitalisation, visits to family clinics and referrals to other clinical specialists in the past year were gathered. In multivariable logistic regression modelling the factors associated with past year psychological IPV exposure were identified, with unemployment or working part-time (*p* = 0.001), a college degree (*p* = 0.038), an intimate relationship of six years or more (*p* = 0.048) and a history of disputes in the intimate relationship (*p* < 0.001) increasing the odds of emotional abuse and explaining 41% of the variance. In females, unemployment (*p* = 0.002) and a history of disputes in the intimate relationship (*p* < 0.001) explained 43% of the variance.

In present study, FDs were asked if they had attended the “Recognizing and Treating Victims of Domestic Violence in Health Care Settings: Guidelines and Training for Health Professionals” training program for improving the competences of health professionals in recognizing and responding to victims of domestic violence. This education and training for health professionals took place between September 2015 and March 2016 at the Medical Chamber of Slovenia and was funded by Norway Grants [18]. Thirty-seven responded affirmatively and were considered properly empowered and competent to meet this study’s requirements. The FDs were provided with written instructions on their approach to the patients, including an invitation letter to the patients, eligibility criteria and on data collection, i.e., the depression section of the Composite International Diagnostic Interview (CIDI) [16], semi-structured interview forms and medical charts review forms; they were already familiar with the latter since the same forms were used in the 2013 Psychological IPV study [9].

#### 2.1.1. The First Phase: Identification of Patients

The participating FDs were asked to identify the patients from the 2013 study and invite them to contact the designated family clinic and make an appointment. The first phase of data collection was planned for January to March 2017 but was actually carried out during March and April, when the FDs, who work in family practices all over the country, interviewed 283 patients (of 471 invited) who attended and were willing to participate. Other eligibility criteria for this phase were age and the absence of dementia or even mild cognitive impairment. In the invitation letter to patients, the aim of the study was explained as being a follow-up on people’s wellbeing after a four-year gap and the subjects were also informed that participation was not obligatory. Those willing to participate made appointments and were scheduled for an interview at the patients’ convenience within two to seven weeks. This phase of data collection ended on 30 April 2017.

#### 2.1.2. The Second Phase: Interviewing the Patients

The FDs explained to the participants (283 of 471 invited (the response rate being 60.1%)) the associations between exposure to IPV and quality of life. Prior to this phase, the FDs revised the elements to be covered in the semi-structured interviews. After the introduction to the IPV topic, 246 patients signed a written consent form, while 37 decided against participation and were not interviewed any further. The interviewing phase lasted until the end of April; during the 246 interviews, 72 patients revealed they had not lived together with an intimate partner in their adulthood or at least in the past decade They explained that they were living either with their parents, their children, both or alone. Those who had never shared a household with an intimate partner were excluded from further analysis. During the interview, a further 13 patients declared they were not willing to complete the self-assessment scale for depression. As the study aimed to analyse associations between self-rated depression and possible IPV exposure, patients without a correctly completed depression scale sheet were excluded from the analysis regardless of their IPV exposure; however, they were offered help and assistance, similarly to the previous study. After the self-administration of the SDS, a nurse calculated the total score and the FDs, who were instructed to evaluate the screening results, interviewed patients who scored above 30 points, using the depression section of the CIDI [16].

#### 2.1.3. The Third Phase: Auditing Patients’ Medical Charts

The auditing of patients’ medical charts was carried out using The Domestic Violence Exposure Medical Chart Check List [19,20], described in detail elsewhere. All medical conditions were checked from 2012, as requested in the 2013 IPV study, up to the present time, covering the whole five years. Only those persisting at least for three years, either three consecutive years or three times in a specified period of inspection, were marked. Given that the FDs participated in the 2013 study, they were familiar with the list. However, they were provided with additional clarifications regarding several variables which were found to be ambiguous in the previous study [9]: incapacity for work was defined as the inability of the victim, due to their exposure to IPV, to perform the normal duties of work in their job or at the post occupied; reduced physical functioning was clarified strictly as a functional and consequently psychosocial impairment i.e., not attending any recreational physical activities, neglecting one’s hobbies and interests, and/or omitting attendance at social functions and duties; a dysfunctional family was marked as a family in which conflict, lack of empathy and support, misbehaviour, and/or child neglect or abuse on the part of individual parents occurred; and sick leave assessment was taken into account only as absence from work measured in days due to the consequences of family violence.

#### 2.1.4. Participants: Study Sample

**Figure 1:**
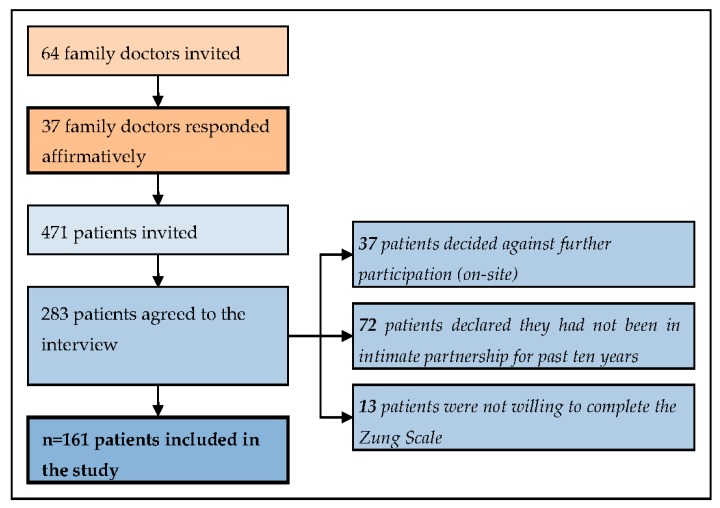
The drop-out data.

In total, the medical charts of 161 participants, male and female, who declared that they had been living with their intimate partners at least during the past decade were reviewed. The drop-out data of the recruitment process are presented in Figure 1.

The mean age of all the participants (*n* = 161) was 51.1 ± 16.8 (range 22–92 years), while the mean age of the partner (of those patients who were married or were living in an intimate relationship, *n* = 115) was 49.7 ± 16.1 (range 20–93 years). The mean value for Zung SDS was 33.2 ± 9.3. In total, 65 (40.4%) participants were exposed to IPV at any time in their adult life.

The National Medical Ethics Committee of the Republic of Slovenia approved the protocol of the study (document number 77/01/15 from 19 January 2014).

### 2.2. Measures

The short form of A Domestic Violence Exposure Questionnaire was developed in previous studies in Slovenian primary care [19,20,21] and used as an exploration tool, consisting of specific questions with comprehensive, behaviourally-defined descriptions of interpersonal violence events which were to be considered within the patients’ whole adult life. An adult was defined by the right to vote at the age of 18 years, regardless of financial, emotional or other types of autonomy in the individual. The questions were worded as follows: *Do you feel safe at home? Do you feel accepted, respected and loved in your intimate relationship? Have you been humiliated, subjected to threats, insult or intimidation, or in any way emotionally affected by your intimate partner? Does your partner talk down to you? Has he/she demeaned or insulted you or made you feel ashamed? Has he/she screamed or cursed at you? Has he/she threatened you with physical harm?* The patients were not asked to specify whether this had happened in the previous year and/or any number of years preceding the survey, or with a current or former intimate partner; for the aim of this study, it was important for patients to disclose if they had ever been exposed to IPV in their adult lives. However, the FDs noted answers referring to events in the past year.

Additionally, the patients were asked whether, aside from the aforementioned behaviours, they had endured any patterns of physical activity that had harmed or might have harmed them, e.g., slapping, kicking, pushing, being forced into sexual activities. The interviews were closed by asking *If such a thing has happened, have you been thinking about doing something about it? Do you want me to help?* The remaining data collected were on gender, age, number of children, marital status and number of divorces and place of residence.

The Zung Self-Rating Depression Scale (SDS) [22] was used to assess depression. In the 2013 study [9], depression was evaluated using the using the depression section of the Composite International Diagnostic Interview, which provided psychiatric diagnoses based on symptoms experienced in the last six months, according to the ICD-10 criteria. Several risk factors, intrinsic either to the individual or to the social context, were included. However, the quality control of these interviews was not strictly performed and questions about interviewers’ competencies related to bias appeared. The Zung scale has been widely used in Slovenia [23] so it was decided on, regardless of better measures being available, since we wanted to be able to compare results with previous findings. The Zung scale consists of 20 statements, 10 of them reversed and each one is scored from 1 to 4, making the range of the completed scale from 20 to 80. All the participants completed the Zung SDS and a depressive score was calculated and interpreted as follows: less than 39 points, no depression; between 40 and 49 points, marginal depression; and above 50 points, depression was indicated. Cronbach’s alpha was 0.79. The cut-off score of 40 points was calculated based on the CIDI, which was performed after the SDS administration. The FDs categorised the CIDI results as depression indicated and depression not indicated.

After the interviews took place, the FDs reviewed the patients’ medical charts, replicating the procedure from previous IPV studies in Slovenian family medicine [9,17,21], focusing on the health-related associations of exposure to IPV and data on the patients’ wider life context at both the personal and relationship level, as reported by the patient in previous years. Health consequences were later categorized into three groups: physical, sexual and reproductive and psychological; this procedure has been described in depth elsewhere [9].

Aiming to follow up the 2013 study results, sick leave (in episodes and days), hospitalization (in episodes and days), visits to the family clinic and referrals to other clinical specialists in the past year were reviewed and are presented in Table 1. ‘Frequent’ was defined as within the top 10 percentile in a time frame of one year for each characteristic. The utilization of healthcare services was additionally analysed concerning self-rated depression.

### 2.3. Data Analysis

In the data analysis, frequencies and percentages were used to describe the main characteristics of this study sample (Table 2).

In power calculation, for the design effect the calculation equation 1 + (m − 1) × ρ given by Donner et al. [24] was used. The intra-cluster correlation coefficient (ρ) was taken from a large sample study by Smeeth et al. [25] for cluster randomized trials and surveys at the primary care clinic level (a maximum value observed of 0.05). The mean cluster size (m) was 4.4 ± 1.7 patients (range 3–8) and the design effect was equal to 1.17. Consequently, the effective sample size was calculated at *n* = 137 (the actual sample size divided by the design effect). Considering sample size tables by Hsieh [26], a total of 137 cases was determined to have 70% power to detect a significant association for logistic regression (using alpha of 0.05 and a medium odds ratio of about 2.5 to 1 [27]).

The patients were compared with regard to self-rated depression (≤40 (no depression) and >40 (borderline depression or depression)) and also the IPV exposure during their adult life (‘an IPV experience’ vs. ‘never’); see Table 3 and Table 4. Multivariable logistic regression was used to model demographic characteristics in association with self-rated depression (Table 5) and afterwards another multivariable logistic modelling was performed, also including variables derived from medical chart analysis, i.e., divorce in the past (yes/no); history of conflict or dispute in the family (yes/no); dysfunctional family relations (yes/no); physical condition: incapacity to work; physical condition: reduced physical functioning; sexual and reproductive consequences of IPV exposure: gynaecological disorders; and exposure to IPV at any time (yes/no). The results were presented by adjusted odds ratios with 95% confidence intervals. Statistical analysis was performed by IBM SPSS 20.0 software (IBM Corp., Armonk, NY, USA) and *p* < 0.05 was set as the level of statistical significance.

## 3. Results

### 3.1. Demographic Characteristics of Patients

The demographic characteristics of the sample are presented in Table 2. Of the sample, 40.4% (*n* = 65) people had been exposed to IPV at some time in their adulthood (58 women and 7 men).

### 3.2. Self-Rated Depression in Patients Exposed to IPV

Of the patients exposed to IPV (*n* = 65), 24 (36.9%) scored >40 on the SDS. Overall the participants scored 33.2 ± 9.3 on the SDS; minimum 20, maximum 57. More results on self-rated depression in IPV patients are described in Table 3.

### 3.3. Frequent Use of Health Care Services by Participating Patients during the Past Year

There were no statistically significant differences found between people exposed to any type of IPV and those who did not report such experiences (Table 1).

### 3.4. Patients’ Medical Charts Review—Summary

The summary of the patients’ medical charts is presented in Table 4. Statistically significant differences in physical status were identified between the patients who were—according to the SDS—depressed and those who were not, with regard to incapacity to work (*p* < 0.001), muscle inflammations, i.e., myalgia, muscle soreness or musculoskeletal pain (*p* < 0.001) and reduced physical functioning (*p* < 0.019). Gynaecological disorders were significantly more frequent in the depressed patients (*p* = 0.015), as well as several items in the psychological behavioural status, including the state of depression and/or generalized anxiety disorder (*p* < 0.001), eating and sleeping disorders (*p* < 0.001), feelings of shame and guilt (*p* = 0.001), phobias and panic attacks (*p* = 0.012), physical inactivity (*p* = 0.001), low self-esteem (*p* < 0.001), psychosomatic disorders (*p* < 0.001) and smoking (*p* = 0.027), all assessed or diagnosed by practicing FDs from the year prior to the 2013 survey [9] (March 2012) until April or at the latest December 2016, if persistent for at least three years, i.e., three consecutive years or three times during this period.

According to the 40.4% IPV exposure at any time in patients in Table 4, statistically significant differences in physical status were identified between the patients who were exposed to IPV and those who were not, with regard to chronic pain syndrome (*p* = 0.021), incapacity to work (*p* = 0.036), muscle inflammations (myalgia, muscle soreness or musculoskeletal pain; *p* = 0.022), gastrointestinal disorders (*p* = 0.003), irregularities in bowel functioning (*p* < 0.001) and reduced physical functioning (*p* = 0.009). Gynaecological disorders were significantly more frequent in IPV exposed patients (*p* = 0.011), as were genital tract infections (*p* = 0.002), as well as several items in the psychological behavioural status, including the state of depression and/or generalized anxiety disorder (*p* < 0.001), eating and sleeping disorders (*p* < 0.001), phobias and panic attacks (*p* = 0.022), low self-esteem (*p* < 0.001) and psychosomatic disorders (*p* < 0.001); all were assessed or diagnosed by practising FDs.

### 3.5. Associations between Self-Rated Depression and Bio-Psycho-Social Characteristics in Patients: Logistic Regression Modelling

In the regression modelling process, the associations between self-rated depression and the characteristics of patients were explored. Exposure to emotional (aOR 3.04, 95% CI 1.12–8.27, *p* = 0.029) and to physical violence (aOR 4.69, 95% CI 1.42–15.49, *p* = 0.011) were identified as risk factors, explaining 23% of the variance (Nagelkerke R^2^ = 0.229, *p* = 0.028). The results are presented in Table 5.

When analysing only female patients, there was almost the same division of explained variance (Nagelkerke R^2^ = 0.227, *p* = 0.084) and the two risk factors identified (exposure to emotional (aOR 3.41, 95% CI 1.13–10.37, *p* = 0.030) and to physical violence (aOR 5.14, 95% CI 1.46–18.09, *p* = 0.011)).

### 3.6. Associations between Self-Rated Depression, the Bio-Psycho-Social Characteristics and Medical Charts Review Summary Patients: Logistic Regression Modelling

Additionally, the logistic regression modelling was broadened, also taking into account data gathered in the process of patients’ medical charts review and performed to explore self-rated depression and its associations in all patients and separately in female patients. In females, divorce in the past (aOR 8.80, 95% CI 1.21–63.91, *p* = 0.032), dysfunctional family relationships (aOR 25.30, 95% CI 3.12–205.37, *p* = 0.02) and a history of incapacity to work identified by the GP in the patient’s medical chart review (aOR 22.16, 95% CI 2.71–181.08, *p* = 0.004) increased the odds of depression in female patients, with regression modelling explaining 65% of the variance (Nagelkerke R^2^ = 0.655, *p* < 0.001). The model which included both female and male patients explained 66% of the variance (Nagelkerke R^2^ = 0.657, *p* < 0.001) with divorce in the past (aOR 6.11, 95% CI 1.12–33.48, *p* = 0.037), dysfunctional family relationships (aOR 19.29, 95% CI 3.13–118.87, *p* = 0.001) and a history of incapacity to work identified by the GP in the patient’s medical chart review (aOR 18.94, 95% CI 3.26–109.97, *p* = 0.001) increased the odds of depression in patients.

## 4. Discussion

The prevalence of depression was found to be associated with exposure to IPV anytime in the adult life and women were found to be more likely to be victimized (Table 3 and Table 5). The physical health consequences associated with exposure to IPV were incapacity to work, reduced physical functioning and muscle inflammation, while the psychological consequences included smoking, panic attacks and anxiety, eating disorders, feelings of shame and guilt and low self-esteem (Table 1, Table 3 and Table 4).

Depression in family practice attendees in Slovenia was found to be significantly associated with female gender, chronic conditions, chronic pain and age above 45 years in a study by Klemenc Ketiš [23]. The Self-Rated Depression Scale was used in that study as well as in the present one and the difference in the overall rate of depression detected in the participants (15.2% vs. 27.8%) could be attributed to the different mean age of the participants (44.2 vs. 51.1 years). Nearly 37% of all the participants in our study who had been exposed to violence at any time in their adult lives fulfilled the criteria for depression according to the SDS (Table 3), compared to 21.7% of depressive patients with chronic conditions and to 23.2% of those with chronic pain [23]. Past exposure to IPV was not only associated with depression but also with suicide attempts, according to a meta-analysis by Devries et al. [28]. Since IPV has been claimed to trigger a whole range of depressive symptoms, this might explain why the rate of depression in the participants exposed to IPV was higher than in those with chronic conditions and chronic pain [23]. Another study by Selič et al. in 2013, in the same environment, detected an 89.5% rate of depression and general anxiety disorder in the participants exposed to IPV [9]. A more recent publication by the same author introduced rates of depression in elderly people in Slovenia, reporting depression to be significantly associated with female gender, age, years of education, smoking, number of concurrent diseases and self-rated health regarding heart, blood vessels, musculoskeletal system and mental health but comparably to our results (Table 2)—not with marital status or alcohol consumption [29]. As the mean age of the participants was 75.1 ± 6.2 years, the empty nest stage of the family cycle, the remoteness of possible exposure to IPV and possibly living alone may diminish the influence of partner interaction on depression. This finding is congruent with the research of Bonomi et al. [30,31], which found that recent exposure to physical and/or sexual IPV increased the risk of depressive and severe depressive symptoms. The authors emphasized that more pronounced adverse health effects were identified in women with recent (vs. remote) exposure to IPV. Our findings are comparable with those in IPV survivors [30,31]: reported exposure to partner violence and physical violence in both the past year and also at any time in adult life were found to be a statistically significant risk factors for current, self-rated depression (Table 3) and also for generalized anxiety disorder and the prevalence of depression (Table 4). Dysfunctional family relationships were found to be significantly associated with the prevalence of depression in those exposed to IPV (*p* = 0.001); according to Felitti, inquiry into depression, past abuse and past or present dysfunctional family life should be added to the current clinical evaluation [32]. Poor family functioning has long been recognized as a factor associated with IPV [33,34], either as the cause or the consequence of the IPV, similar to our findings with IPV related depression in all patients and also separately in female patients (*p* = 0.002). The association between IPV, poor family functioning and divorce in the past (*p* = 0.032) and consequent depression in participants in this study is concordant with that described as complex and multidirectional by others [1,11,35].

Several authors report that victims of violence seek medical assistance more often than violence free individuals [11,36,37], which was not confirmed in our study (Table 1). However, the utilisation of healthcare services, i.e., sick leave (46 or more days) and visits to the family clinic (16 or more) were more frequent in depressed attendees, all patients and females only (*p*_all_ = 0.020, *p*_females_ = 0.032; *p*_all_ = 0.007, *p*_females_ = 0.005, respectively); while hospitalization (1 or more days) was more likely to be used by this study sample (*p* = 0.027) but by not females separately (Table 1). Evidencing the underlying cause of a specific sick leave as a consequence of IPV may be challenging and unclear and could contribute to the non-significant difference in the sick leave of individuals exposed to IPV versus those who were not (Table 1). In extreme cases, the IPV and consequent health issues do not only result in incapacity for work but also cause permanent loss of working capability, debilitation, deterioration of quality of life and premature death [6,10,38]. In accordance with these data, incapacity to work and reduced physical functioning in our study were the most significant physical consequences associated with self-rated depression (Table 4) and also with IPV exposure in adult life (Table 4). A longitudinal study on a representative sample of Slovenian family medicine patients [39] brought good insight into the quality of life related factors. Since the sample was representative, it is important to be aware of factors significantly and consistently associated with a better mental component score of quality of life, i.e., social support, satisfactory circumstances in patients` household (absence of violence) and absence of anxiety.

### 4.1. Limitations of the Study

The sample in this study could not be considered as representative of a family medicine attendees’ population [40], since there were more women (76.4% vs. 54.8%) and the level of education was much higher (46.6% vs. 11.3% people with college degree or above, 9.3% vs. 41.0% with elementary school) but the mean age was almost the same (51.1 ± 16.8 years vs. 51.7 ± 19.0 years). Yet there are not so many noticeable differences in comparison with the 2013 study [9], i.e., in the present study there were also more women (76.4% vs. 61.3%) and the mean age of this study’s participants was slightly older (51.1 ± 16.8 years vs. 47.4 ± 16.1 years), while the level of education appears similar (46.6% vs. 45.7% people with college degree or above, 9.3% vs. 11.1% with elementary school). Given the females prevailing in the sample (76.4%), the findings of this study should be used with caution, without emphasizing gender specificities.

The opportunistic sampling method, i.e., the sample size of 161 out of 471 invited patients, may not have reflected the diversity of the Slovenian community and might have affected the IPV exposure prevalence, which was found to be 40.4% for the whole adult life. It is therefore of the utmost importance that another study of lifetime IPV exposure in Slovenian family medicine attendees be performed soon, regardless of possible mental health consequences as a special focus of interest.

Reliance on self-reported data and social desirability bias may have both impacted on results, given that IPV is still considered as a private and/or too sensitive an affair for many people to intervene in Slovenia [41]. The burden of somatic co-morbidity was shown to be smaller than the impact of psychosocial determinants in a study on a representative sample of family practice attendees [16], emphasizing the social stigma of mood disorders. As explained in the Method section, there were 13 participants unwilling to complete the SDS. Given that the detection and successful treatment of depression and anxiety have already been recognized as facilitating factors with a potential to lead to improved quality of life in family medicine attendees some time ago [40] and consequently FDs were instructed to be alert for the early onset of these conditions, apparently more efforts should be make in awareness-raising in FDs.

The cross-sectional design, known as inherently limited, together with part of the obtained data being self-reported, could have raised hindrances about the potential for method variance to account for this study’s findings. Since the social environment related phenomenon, i.e., the IPV exposure being studied could have been assessed only by asking patients to report their experience or perception, it was of great advantage that the research design incorporated medical records to assemble the exact health-related data (Table 4). Except for the self-reported data on IPV and depression, there were several characteristics followed through the longer period of time (from March 2012 to December 2016 at the latest) reviewed by the FDs in the patients’ medical history, e.g., results of the Composite International Diagnostic Interview conducted in 2013 study [9], physical, sexual and reproductive IPV related health consequences and psychological and behavioural status, which in our opinion alleviated the potential effects of method variance. The partition of explained variance in multivariable modelling grew evidently higher when these data was added (R^2^ = 0.657, *p* < 0.001; R^2^_(females)_ = 0.655, *p*_(females)_ < 0.001) to illustrate the associations between self-rated depression, bio-psycho-social characteristics of patients and their medical charts review summary.

### 4.2. Implications for Future Research and Family Practice

Although only exposure to emotional and physical IPV, both being self-reported, identified as being associated with depression (Table 5), the data was collected in a large number of settings, which hopefully reduced the non-response bias. However, future research should be more focused in following up the initial cohort of patients, given that for this study purpose the cohort was identified with less difficulty than expected, while the FDs had undergone a thorough capacity building process in the field of IPV exposure related concepts, both practical and theoretical.

For the first time in Slovenian family medicine, a clear indication of the association of IPV exposure with depression was found, in spite of the barriers on the side of Slovenian FDs detected in previous studies [41].

We were unable to avoid a high drop-out rate compared to the previous study [9] (Figure 1), so rethinking this future study plan, together with the need to evaluate the 40.4% lifetime IPV exposure prevalence, would be beneficial.

## 5. Conclusions

The prevalence of depression in patients who were exposed to IPV at any time in their adult life was significantly more likely than in non-IPV exposed patients (*p* < 0.001); in those who were exposed to IPV, almost 37% (36.92% (*n* = 24)) were diagnosed as depressed. For this reason, FDs detecting depression in their patients will be advised to explore the possibility of exposure to IPV as a contributing factor and vice versa. Practising FDs should be aware of the overlapping effect of symptoms, e.g., incapacity to work, depression and anxiety in patients. Therefore, those lacking satisfactory household circumstances, regardless of a clear IPV exposure revealed, should be recognized and empowered. The assessment of two out of five adult family medicine attendees who might have been exposed to IPV strengthens this recommendation.

## Figures and Tables

**Table 1:** The utilization of healthcare services concerning IPV exposure and self-rated depression in the last 12 months.

	Top 10 Percentiles	Violence Any Time in Adult Life		* p	Violence Any Time in Adult Life (Female Patients)		p *	Self-Rated Depression		p *	Self-Rated Depression (Female Patients)		p *
		No *n* = 96 (%)	Yes *n* = 65 (%)		No *n* = 65 (%)	Yes *n* = 58 (%)		No *n* = 127 (%)	Yes *n* = 34 (%)		No *n* = 92 (%)	Yes *n* = 31 (%)	
Sick leave (episodes)	3 or more	16 (16.7)	9 (13.8)	0.822	12 (18.5)	6 (10.3)	0.297	18 (14.2)	7 (20.6)	0.250	11 (12.0)	7 (22.6)	0.125
Sick leave (days)	46 or more	5 (5.2)	5 (7.7)	0.504	5 (7.7)	4 (6.9)	1.000	5 (3.9)	5 (14.7)	0.020	4 (4.3)	5 (16.1)	0.032
Hospitalization (episodes)	1 or more	7 (7.3)	7 (10.8)	0.551	6 (9.2)	6 (10.3)	1.000	8 (6.3)	6 (17.6)	0.080	7 (7.6)	5 (16.1)	0.294
Hospitalization (days)	1 or more	6 (6.3)	7 (10.8)	0.382	5 (7.7)	6 (10.3)	0.756	7 (5.5)	6 (17.6)	0.027	6 (6.5)	5 (16.1)	0.134
Visits to family clinic	16 or more	13 (13.5)	6 (9.2)	0.460	10 (15.4)	6 (10.3)	0.426	10 (7.9)	9 (26.5)	0.007	7 (7.6)	9 (29.0)	0.005
Referrals to other clinical specialists	5 or more	11 (11.5)	9 (13.8)	0.809	8 (12.3)	8 (13.8)	1.000	14 (11.0)	6 (17.6)	0.382	12 (13.0)	4 (12.9)	1.000

* Fisher’s exact test.

**Table 2:** Demographics characteristics and IPV exposure of the sample.

	Total *n* (%)	IPV Exposure *n* (%)		*p*
	*n* = 161 (%)	*n* = 96 (%)	Yes *n* = 65 (%)	
**Gender**				0.002 *
Male	38 (23.6)	31 (32.3)	7 (10.8)	
Female	123 (76.4)	65 (67.7)	58 (89.2)	
**Age**				0.990 #
29 years or less	18 (11.2)	11 (11.5)	7 (10.8)	
30–59 years	91 (56.6)	54 (56.3)	37 (56.9)	
60 years or more	52 (32.3)	31 (32.3)	21 (32.3)	
**Married or living in an intimate relationship**				0.155 *
No	46 (28.6)	23 (24.0)	23 (35.4)	
Yes	115 (71.5)	73 (76.0)	42 (64.6)	
**Divorce in the past**				0.006 *
No	125 (77.6)	82 (85.4)	43 (66.2)	
Yes	36 (22.4)	14 (14.6)	22 (33.8)	
**Age of the children**				0.015 *
Less than 15 years	69 (42.9)	49 (51.0)	20 (30.8)	
15 years or more	92 (57.1)	47 (49.0)	45 (69.2)	
**Living in urban setting**				1.000*
No	27 (16.8)	16 (16.7)	11 (16.9)	
Yes	135 (83.2)	80 (83.3)	54 (83.1)	
**Employment status**				0.086 #
Unemployed or working part time	13 (8.1)	4 (4.2)	9 (13.8)	
Regularly employed	96 (59.6)	60 (62.5)	36 (55.4)	
Retired	52 (32.3)	32 (33.3)	20 (30.8)	
**Level of education**				0.223 #
Elementary school	15 (9.3)	6 (6.3)	9 (13.8)	
High school	71 (44.1)	42 (43.8)	29 (44.6)	
College degree or more	75 (46.6)	48 (50.0)	27 (41.5)	
**Monthly income per family member**				0.025 *
Less than 900 EUR	84 (52.2)	43 (44.8)	41 (63.1)	
900 EUR or more	77 (47.8)	53 (55.2)	24 (36.9)	

* Fisher’s exact test, # chi-square test.

**Table 3:** Self-rated depression in patients exposed to IPV according to their SDS score.

	≤40 (No Depression) *n* = 127 (%)	>40 (Borderline Depression or Depression) *n* = 34 (%)	p *
Emotional violence in the last 12 months (*n* = 46)	29 (22.8)	17 (50.0)	0.003
Physical violence at any time in adult life (*n* = 28)	15 (11.8)	13 (38.2)	0.001
Emotional violence at any time in adult life (*n* = 61)	39 (30.7)	22 (64.7)	0.001
Violence at any time in adult life (*n* = 65)	41 (32.3)	24 (70.6)	<0.001

* Fisher’s exact test, SDS = Zung Self-Rated Depression Scale.

**Table 4:** Patients’ medical charts review summary taking into account self-rated depression and IPV exposure in patients.

	Depression According to SDS *n* (%)		IPV Exposure *n* (%)			
	No Depression *n* = 127 (%)	Depression *n* = 34 (%)	cOR (95%CI), p *	No IPV *n* = 96	IPV *n* = 65	cOR (95%CI), p *
**Physical status**						
Injuries: head, thoracic and abdominal area (*n* = 13)	11 (8.9)	2 (6.1)	0.66 (0.14–3.13), 1.000	8 (8.3)	5 (7.7)	0.92 (0.29–2.94), 1.000
Scratches and bruises (*n* = 30)	24 (19.4)	6 (17.6)	0.92 (0.34–2.47), 1.000	15 (15.6)	15 (23.1)	1.62 (0.73–3.60), 0.302
Chronic pain syndrome (*n* = 132)	100 (81.5)	31 (91.2)	2.79 (0.79–9.83) 0.204	73 (76.0)	59 (90.8)	3.10 (1.18–8.11), 0.021
Incapacity to work (*n* = 47)	26 (21.0)	21 (61.8)	6.28 (2.78–14.18), <0.001	22 (22.9)	25 (38.5)	2.10 (1.05–4.19), 0.036
Muscle inflammation (*n* = 93)	67 (54.0)	26 (76.5)	2.91 (1.22–6.92), 0.019	48 (50.0)	45 (69.2)	2.25 (1.16–4.36), 0.022
Bone fractures (*n* = 31)	28 (22.8)	3 (8.8)	0.34 (0.10–1.20), 0.089	18 (18.8)	13 (20.0)	1.08 (0.49–2.40), 0.842
Gastrointestinal disorders (*n* = 99)	74 (60.2)	25 (73.5)	1.99 (0.86–4.61), 0.167	50 (52.1)	49 (75.4)	2.82 (1.41–5.63), 0.003
Irregularities in bowel functioning (*n* = 66)	47 (38.2)	19 (55.9)	2.16 (0.98–4.64), 0.078	28 (29.2)	38 (58.5)	3.42 (1.76–6.62), <0.001
Lacerations and cuts (*n* = 19)	15 (12.2)	4 (11.8)	0.99 (0.31–3.22), 1.000	11 (11.5)	8 (12.3)	1.08 (0.41–2.86), 1.000
Eye injuries (*n* = 3)	3 (2.4)	0 (0.0)	0.52 (0.03–10.22), 1.000	1 (1.0)	2 (3.1)	3.02 (0.27–33.97), 0.566
Reduced physical functioning (*n* = 39)	20 (16.1)	19 (55.9)	6.78 (2.96–15.52), <0.001	16 (16.7)	23 (35.4)	2.74 (1.31–5.74), 0.009
**Sexual and reproductive status**						
Gynaecological disorders (*n* = 55)	37 (29.8)	18 (52.9)	2.73 (1.26–5.94), 0.015	25 (26.0)	30 (46.2)	2.43 (1.25–4.75), 0.011
Infertility (*n* = 8)	7 (5.6)	1 (2.9)	0.52 (0.06–4.37), 1.000	6 (6.3)	2 (3.1)	0.48 (0.09–2.44), 0.476
Genital tract infections (*n* = 70)	51 (41.1)	19 (55.9)	1.89 (0.88–4.05), 0.172	32 (33.3)	38 (58.5)	2.81 (1.47–5.40), 0.002
Complicated pregnancies/spontaneous abortions (*n* = 25)	18 (14.6)	7 (20.6)	1.71 (0.65–4.51), 0.430	11 (11.5)	14 (21.5)	2.32 (0.99–5.44), 0.119
Sexual dysfunctions (*n* = 1)	0 (0.0)	1 (2.9)	11.42 (0.45–286.69), 0.215	1 (1.0)	0 (0.0)	0.49 (0.02–12.12), 1.000
Sexually transmitted diseases, including HIV/AIDS (*n* = 10)	7 (5.6)	3 (8.8)	1.65 (0.41–6.79), 0.449	6 (6.3)	4 (6.2)	0.98 (0.27–3.63), 1.000
Unplanned/unwanted pregnancies (*n* = 1)	0 (0.0)	1 (2.9)	11.42 (0.45–286.69), 0.215	0 (0.0)	1 (1.5)	4.49 (0.18–111.90), 0.404
**Psychological and behavioural status**						
Abuse of alcohol and drugs (*n* = 5)	5 (4.0)	0 (0.0)	0.32 (0.02–5.98), 0.586	2 (2.1)	3 (4.6)	2.72 (0.37–14.00), 0.394
Depression and/or generalized anxiety disorder (*n* = 79)	46 (37.4)	33 (97.1)	58.11 (7.69–438.97), <0.001	34 (35.4)	45 (69.2)	4.10 (2.09–8.04), <0.001
Eating and sleeping disorders (*n* = 81)	50 (41.0)	31 (91.2)	15.91 (4.62–54.85), <0.001	35 (36.5)	46 (70.8)	4.22 (2.14–8.30), <0.001
Feelings of shame and guilt (*n* = 12)	4 (3.3)	8 (23.8)	9.46 (2.65–33.78), 0.001	4 (4.2)	8 (12.3)	3.23 (0.93–11.21), 0.069
Phobias and panic attacks (*n* = 30)	18 (14.6)	12 (35.3)	3.30 (1.40–7.86), 0.012	12 (12.5)	18 (27.7)	2.68 (1.19–6.04), 0.022
Physical inactivity (*n* = 50)	31 (25.2)	19 (55.9)	3.92 (1.78–8.63), 0.001	30 (31.3)	20 (30.8)	0.98 (0.49–1.93), 1.000
Low self-esteem (*n* = 33)	13 (10.6)	20 (58.8)	12.53 (5.13–30.56), <0.001	14 (14.6)	19 (29.9)	2.42 (1.11–5.27), 0.029
Post-traumatic stress disorder (*n* = 28)	18 (14.6)	10 (29.4)	2.52 (0.94–6.15), 0.073	15 (15.6)	13 (20.0)	1.38 (0.61–3.14), 0.528
Psychosomatic disorder (*n* = 107)	74 (60.2)	33 (97.1)	23.64 (3.13–178.26), <0.001	57 (59.4)	50 (76.9)	2.28 (1.13–4,62), 0.027
Smoking (*n* = 30)	28 (22.8)	2 (5.9)	0.22 (0.05–0.98), 0.027	15 (15.6)	15 (23.1)	1.62 (0.73–3.60), 0.302
Suicidal behaviour and self-harm (*n* = 2)	1 (0.8)	1 (2.9)	3.82 (0.23–62.68), 0.387	1 (1.0)	1 (1.5)	1.48 (0.91–24.17), 1.000
Unsafe sexual behaviour (*n* = 1)	1 (0.8)	0 (0.0)	1.22 (0.05–30.67), 1.000	1 (1.0)	0 (0.0)	0.49 (0.02–12.12), 1.000

* Fisher’s exact test, SDS = Zung Self-Rated Depression Scale, IPV = intimate partner violence, cOR = crude odds ratio, 95% CI = 95% confidence interval.

**Table 5:** Associations between self-rated depression and bio-psycho-social characteristics in patients.

	Depression According to SDS *n* (%)		aOR	(95% CI)		*p*
	No *n* = 127	Yes *n* = 34				
**Gender**						
Male	35 (27.6)	3 (8.8)	Ref	-	-	-
Female	92 (72.4)	31 (91.2)	2.17	0.56	8.45	0.264
**Age**						
29 years or less	14 (11.0)	4 (11.8)	Ref	-	-	-
30–59 years	73 (57.5)	18 (52.9)	0.62	0.13	2.97	0.549
30–60 years or more	40 (31.5)	12 (35.3)	0.91	0.10	8.56	0.935
**Married or living in an intimate relationship**						
No	33 (26.0)	13 (38.2)	Ref	-	-	-
30–Yes	94 (74.0)	21 (61.8)	0.99	0.36	2.70	0.982
**Divorce in the past**						
No	105 (82.7)	20 (58.8)	Ref	-	-	-
30–Yes	22 (17.3)	14 (41.2)	2.50	0.86	7.24	0.092
**Age of the children**						
Less than 15 years	58 (45.7)	11 (32.4)	Ref	-	-	-
30–15 years or more	69 (54.3)	23 (67.6)	0.83	0.22	3.20	0.791
**Living in urban setting**						
No	21 (16.5)	6 (17.6)	Ref	-	-	-
30–Yes	106 (83.5)	28 (82.4)	1.13	0.34	3.78	0.846
**Employment status**						
Unemployed or working part time	8 (6.3)	5 (14.7)	Ref	-	-	-
Regularly employed	78 (61.4)	18 (52.9)	0.58	0.13	2.53	0.467
30–Retired	41 (32.3)	11 (32.4)	0.57	0.08	4.28	0.587
**Level of education**						
Elementary school	10 (7.9)	5 (14.7)	Ref	-	-	-
High school	58 (45.7)	13 (38.2)	0.55	0.11	2.72	0.461
30–College degree or more	59 (46.5)	16 (47.1)	0.74	0.13	4.24	0.733
**Monthly income per family member**						
Less than 900 EUR	63 (49.6)	21 (61.8)	Ref	-	-	-
30–900 EUR or more	64 (50.4)	13 (38.2)	0.84	0.29	2.47	0.752
**Exposure to violence, any time**						
No violence	86 (67.7)	10 (29.4)	Ref	-	-	-
Emotional	28 (22.0)	11 (32.4)	3.04	1.12	8.27	0.029
30–Physical	13 (10.2)	13 (38.2)	4.69	1.42	15.49	0.011

chi-square = 25.711; df = 14; *p* = 0.028; Nagelkerke R^2^ = 0.229, SDS = Zung Self-Rated Depression Scale, aOR = adjusted odds ratio, 95% CI = 95% confidence interval.

## References

[1] Varshney M., Mahapatra A., Krishnan V., Gupta R., Deb K.S. (2016). Violence and mental illness: What is the true story?. J. Epidemiol. Community Health.

[2] Preamble to the Constitution of the World Health Organization as Adopted by the International Health Conference, New York, 19–22 June 1946. https://hero.epa.gov/hero/index.cfm/reference/details/reference_id/80385.

[3] Reisenhofer S., Taft A. (2013). Women’s journey to safety—The Transtheoretical model in clinical practice when working with women experiencing Intimate Partner Violence: A scientific review and clinical guidance. Patient Educ. Couns..

[4] Juninger J., McGuire L. (2004). Psychotic motivation and the paradox of current research on serious mental illness and rates of violence. Schizophr. Bull..

[5] Kishor S., Johnson K. (2004). Profiling Domestic Violence: A Multi-Country Study. Calverton, MD, ORC Macro. http://dhsprogram.com/pubs/pdf/OD31/OD31.pdf.

[6] Coker A.L., Smith P.H., Bethea L., Kong M.R., McKeown R.E. (2000). Physical health consequences of physical and psychological intimate partner violence. Arch. Fam. Med..

[7] Elbogen E.B., Johnson S.C. (2009). The intricate link between violence and mental disorder: Results from the National Epidemiologic Survey on Alcohol and Related Conditions. Arch. Gen. Psychiatry.

[8] Amore M., Menchetti M., Tonti C., Scarlatti F., Lundgren E., Esposito W., Berardi D. (2008). Predictors of violent behavior among acute psychiatric patients: Clinical study. Psychiatry Clin. Neurosci..

[9] Selic P., Svab I., Kopcavar Gucek N. (2014). A cross-sectional study identifying the pattern of factors related to psychological intimate partner violence exposure in Slovenian family practice attendees: What hurt them the most. BMC Public Health.

[10] Coker A.L., Davis K.E., Arias I., Desai S., Sanderson M., Brandt H.M., Smith P.H. (2002). Physical and mental health effects of intimate partner violence for men and women. Am. J. Prev. Med..

[11] World Health Organization (2013). Global and Regional Estimates of Violence against Women: Prevalence and Health Effects of Intimate Partner Violence and Non-Partner Sexual Violence.

[12] Hyde J.S., Mezulis A.H., Abramson L.Y. (2008). The ABCs of depression: Integrating affective, biological, and cognitive models to explain the emergence of the gender difference in depression. Psychol. Rev..

[13] Astbury J., Cabral M. (2000). Women’s Mental Health: An Evidence Based Review.

[14] Bonomi A.E., Anderson M.L., Reid R.J., Rivara F.P., Carrell D., Thompson R.S. (2009). Medical and Psychosocial Diagnoses in Women with a History of Intimate Partner Violence. Arch. Intern. Med..

[15] Golding J. (1999). Intimate partner violence as a risk factor for mental disorders: A meta-analysis. J. Fam. Violence.

[16] Selič P., Svab I., Rifel J., Pavlič D.R., Cerne A., King M., Nazareth I. (2011). The pattern of physical comorbidity and the psychosocial determinants of depression: A prospective cohort study on a representative sample of family practice attendees in Slovenia. Ment. Health Fam. Med..

[17] Selic P., Svab I., Kopcavar Gucek N. (2013). How many Slovenian family practice attendees are victims of intimate partner violence?. A re-evaluation cross-sectional study report. BMC Public Health.

[18] POND Project Recognizing and Treating Victims of Domestic Violence in Slovenia. http://eeagrants.org/News/2015/Recognising-and-treating-victims-of-domestic-violence-in-Slovenia.

[19] Selic P., Pesjak K., Kersnik J. (2011). The prevalence of exposure to domestic violence and the factors associated with co-occurrence of psychological and physical violence exposure: A sample from primary care patients. BMC Public Health.

[20] Kopčavar Guček N., Švab I., Selič P. (2011). The prevalence of domestic violence in primary care patients in Slovenia in a five-year period (2005–2009). Croat. Med. J..

[21] Selič P., Pesjak K., Kopčavar Guček N., Kersnik J. (2008). Dejavniki, ki povečujejo možnost nasilja v družini in iskanje pomoči pri zdravniku družinske medicine. Pilotna študija o nasilju v družini. (Factors that increase likelihood of violence in the family and seeking help from the family practitioner. Pilot study about violence in the family). Zdr. Vestnik.

[22] Zung W.W. (1965). A self-rating depression scale. Arch. Gen. Psychiatry.

[23] Klemenc Ketiš Z. (2009). The presence of anxiety and depression in the adult population of family practice patients with chronic diseases. Zdr. Varst..

[24] Donner A., Birkett N., Buck C. (1981). Randomization by cluster. Sample size requirements and analysis. Am. J. Epidemiol..

[25] Smeeth L., Ng E.S. (2002). Intraclass correlation coefficients for cluster randomized trials in primary care: Data from the MRC trial of the assessment and management of older people in the community. Control Clin. Trials.

[26] Hsieh F.Y. (1989). Sample size tables for logistic regression. Stat. Med..

[27] Rosenthal J.A. (1996). Qualitative descriptors of strength of association and effect size. J. Soc. Serv. Res..

[28] Devries K.M., Mak J., Bacchus L.J., Child J.C., Falder G., Petzold M., Astbury J., Watts C.H. (2013). Intimate Partner Violence and Incident Depressive Symptoms and Suicide Attempts: A Systematic Review of Longitudinal Studies. PLoS Med..

[29] Selič P., Skela-Savič B., Hvalič Touzery S. (2017). Cross-sectional study exploring factors associated with depression in elderly living at home. Continuous Development of Nursing in Society and its Contribution to Health Promotion = Proceedings of Lectures with Peer Review.

[30] Umubyeyi A., Mogren I., Ntaganira J., Krantz G. (2014). Intimate partner violence and its contribution to mental disorders in men and women in the post genocide Rwanda: Findings from a population based study. BMC Psychiatry.

[31] Bonomi A.E., Thompson R.S., Anderson M., Reid R.J., Carell D., Dimer J.A., Rivara F.P. (2006). Intimate Partner Violence and Women’s Physical, Mental, and Social Functioning. Am. J. Prev. Med..

[32] Felitti V.J. (1993). Childhood sexual abuse, depression, and family dysfunction in adult obese patients: A case control study. South. Med. J..

[33] Heise L., Garcia Moreno C., Krug E.G., Dahlberg L.L., Mercy J.A., Zwi A.B., Lozano R. (2002). Violence by intimate partners. World Report on Violence and Health.

[34] Black D.A., Heyman R.E., Smith Slep A.M. (2001). Risk factors for child physical abuse. Aggress. Violent Behav..

[35] Mechanic M.B., Weaver T.L., Resick P.A. (2008). Mental health consequences of intimate partner abuse: A multidimensional assessment of four different forms of abuse. Violence Women.

[36] Dahlberg L.L., Krug E.G., Krug E.G., Dahlberg L.L., Mercy J.A., Zwi A.B., Lozano R. (2002). Violence—A global preventive problem. World Report on Violence and Health.

[37] WHA49.25 Prevention of Violence: A Public Health Priority. Forty-Ninth World Health Assembly Geneva 1996. http://www.who.int/violence_injury_prevention/resources/publications/en/WHA4925_eng.pdf.

[38] Wagner P., Mongan P. (1998). Validating the concept of abuse: Women’s perceptions of defining behaviors and the effects of emotional abuse on health indicators. Arch. Fam. Med..

[39] Cerne A., Svab I., Kersnik J., Selic P. (2013). Did past economic prosperity affect the health related quality of life predictors? A longitudinal study on a representative sample of Slovenian family medicine patients. BMC Public Health.

[40] Svab I., Petek Ster M., Kersnik J., Zivcec Kalan G., Car J. (2005). A cross sectional study of performance of Slovene general practitioners (English summary). Zdr. Varst..

[41] Kopčavar Guček N., Petek D., Švab I., Selič P. (2015). Barriers to Screening and Possibilities for Active Detection of Family Medicine Attendees Exposed to Intimate Partner Violence. Zdr. Varst..

